# Interaction Between Rec8 and Mis4 Is Required for Axis‐Loop Chromatin Formation and Homologous Chromosome Recombination During Meiosis

**DOI:** 10.1111/gtc.70128

**Published:** 2026-06-10

**Authors:** Takeshi Sakuno, Yasuto Murayama, Tokuko Haraguchi, Yasushi Hiraoka

**Affiliations:** ^1^ Graduate School of Frontier Biosciences, the University of Osaka Suita Japan; ^2^ National Institute of Genetics Mishima Japan

## Abstract

The Rec8 cohesin complex is required for the pairing and recombination of homologous chromosomes during meiosis, as well as for the cohesion of sister chromatids. In the fission yeast *Schizosaccharomyces pombe*, we previously identified a *rec8‐F204S* mutant that lost the ability to assemble the axis‐loop chromatin structure without losing sister chromatid cohesion. This mutant showed reduced meiotic recombination, indicating that pairing and recombination of homologous chromosomes require the formation of the axis‐loop chromatin structure mediated by the Rec8 cohesin complex. Loading of the Rec8 cohesin complex onto chromatin is mediated by Mis4 (NIPBL in humans; Scc2 in yeast). In this study, to elucidate the functions of Mis4, we identified a *mis4‐LR* mutant (L1150S and R1159G) that reproduced the phenotypes of the *rec8‐F204S* mutant, which is defective in chromatin axis formation and homologous recombination while retaining sister chromatid cohesion. These mutation sites (Mis4‐L1150, Mis4‐R1159, and Rec8‐F204) are all localized at the interaction surface between Mis4 and Rec8. Biochemical analysis revealed that the Mis4‐LR mutant protein exhibited reduced Rec8‐binding activity. Considering that the *mis4‐LR* mutant phenocopied the *rec8‐F204S* mutant, our results demonstrate that the Mis4‐Rec8 interaction is required for proper formation of Rec8‐dependent meiotic chromosome axis.

## Graphical abstract

We identified a Mis4 cohesin loader mutant that is specifically defective in chromatin axis formation and homologous recombination while retaining normal sister chromatid cohesion. The *mis4‐LR* mutant, carrying the L1150S and R1159G amino acid substitutions, exhibited reduced interaction between Mis4 and Rec8, suggesting a meiosis‐specific role for the Mis4–Rec8 interaction.

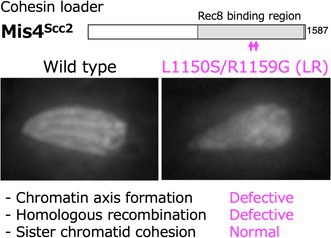

## 1 Introduction

Meiosis is a process of fundamental importance for sexually reproducing eukaryotes, in which one round of DNA replication is followed by two consecutive rounds of chromosome segregation to produce haploid gametes that are inherited by offspring. During this process, homologous chromosomes pair and subsequently undergo recombination. Homologous chromosomes are segregated in the first meiotic division and sister chromatids are segregated in the second meiotic division. Crossovers formed by reciprocal recombination constitute a physical link between homologous chromosomes, ensuring their correct segregation (Zickler and Kleckner 1999; Hunter 2015). Failure of this process is a major cause of miscarriage or developmental disorders caused by chromosome trisomy, such as Down syndrome, in humans (Nagaoka et al. 2012; Webster and Schuh 2017).

The mitotic Rad21 cohesin complex establishes cohesion between a pair of replicated sister chromatids. The cohesin complex consists of four protein components: two Structural Maintenance of Chromosomes (SMC) components, one kleisin component, and one stromal antigen (SA) component (Nasmyth 2001; Mehta et al. 2013; Ishiguro 2019; Sakuno and Hiraoka 2022); these components are called SMC1α, SMC3, RAD21, and SA1 or SA2, respectively, in human cells. Cohesin complexes are also involved in the formation of higher‐order chromatin structures by bringing together distant regions of DNA, for which a DNA loop extrusion model has been proposed (Mizuguchi et al. 2014; Sanborn et al. 2015; Fudenberg et al. 2016; Rao et al. 2017; Davidson et al. 2019; Kim et al. 2019; Davidson and Peters 2021). Cohesin complexes are recruited to chromatin by NIPBL (Mis4 in *S. pombe*) (Higashi et al. 2020; Kurokawa and Murayama 2020; Alonso‐Gil and Losada 2023), and dissociated from chromatin by PDS5 and WAPL (Pds5 and Wpl1 in *S. pombe*) (Ding et al. 2006; Peters and Nishiyama 2012; Tedeschi et al. 2013; Challa et al. 2016; Crawley et al. 2016; Haarhuis et al. 2017; Hong et al. 2019; Silva et al. 2020; Viera et al. 2020).

While the regulated segregation of sister chromatids in the mitotic cell cycle is mediated by the mitotic cohesin complex, meiosis‐specific cohesin variants appear upon entry into meiosis to orchestrate meiosis‐specific chromosome events (Nasmyth 2001; Revenkova and Jessberger 2006). Replacement of the kleisin subunit during meiosis occurs in most organisms, in which the mitotic kleisin RAD21 is replaced by REC8 (Mehta et al. 2013; Nasmyth 2001; Ishiguro 2019; Sakuno and Hiraoka 2022). In addition to sister chromatid cohesion, the meiotic Rec8 cohesin complex is required for pairing and recombination of homologous chromosomes (Klein et al. 1999; Pasierbek et al. 2001; Cai et al. 2003; Ishiguro et al. 2014; Fukuda et al. 2014; Hopkins et al. 2014). Rec8‐dependent chromatin structure is a key determinant of pairing and recombination of homologous chromosomes (Ding et al. 2006, 2016; Sakuno et al. 2022; Sakuno and Hiraoka 2022).

Recent studies using high‐throughput chromosome conformation capture (Hi‐C) clearly revealed that the Rec8 cohesin complex is essential for constructing meiosis‐specific chromatin axis‐loop structures (Schalbetter et al. 2019; Sakuno et al. 2022). Using Hi‐C analysis, we delineated meiotic chromosome structures in the fission yeast *Schizosaccharomyces pombe*, and demonstrated the significance of Rec8‐dependent axis‐loop structures by identifying a *rec8* mutant (*rec8‐F204S*) that is specifically defective in axis‐loop formation and homologous recombination without affecting sister chromatid cohesion (Sakuno et al. 2022).

*S. pombe* Mis4 (NIPBL in human; Scc2 in yeast), a cohesin loader, modulates cohesin functions through its DNA binding, cohesin binding, and ATPase activities (Higashi et al. 2020; Kurokawa and Murayama 2020; Wei et al. 2025). However, it remained unknown how Mis4 is involved in cohesin functions during meiosis. In this study, we isolated a *mis4* mutant that exhibited defects in chromatin axis formation and homologous recombination while retaining sister chromatid cohesion, similarly to the *rec8‐F204S* mutant. Biochemical analysis of the mutant demonstrated the role of Mis4 in meiosis through its interaction with Rec8.

## 2 Results

### 2.1 Isolation of *mis4* Mutants

**FIGURE 1:**
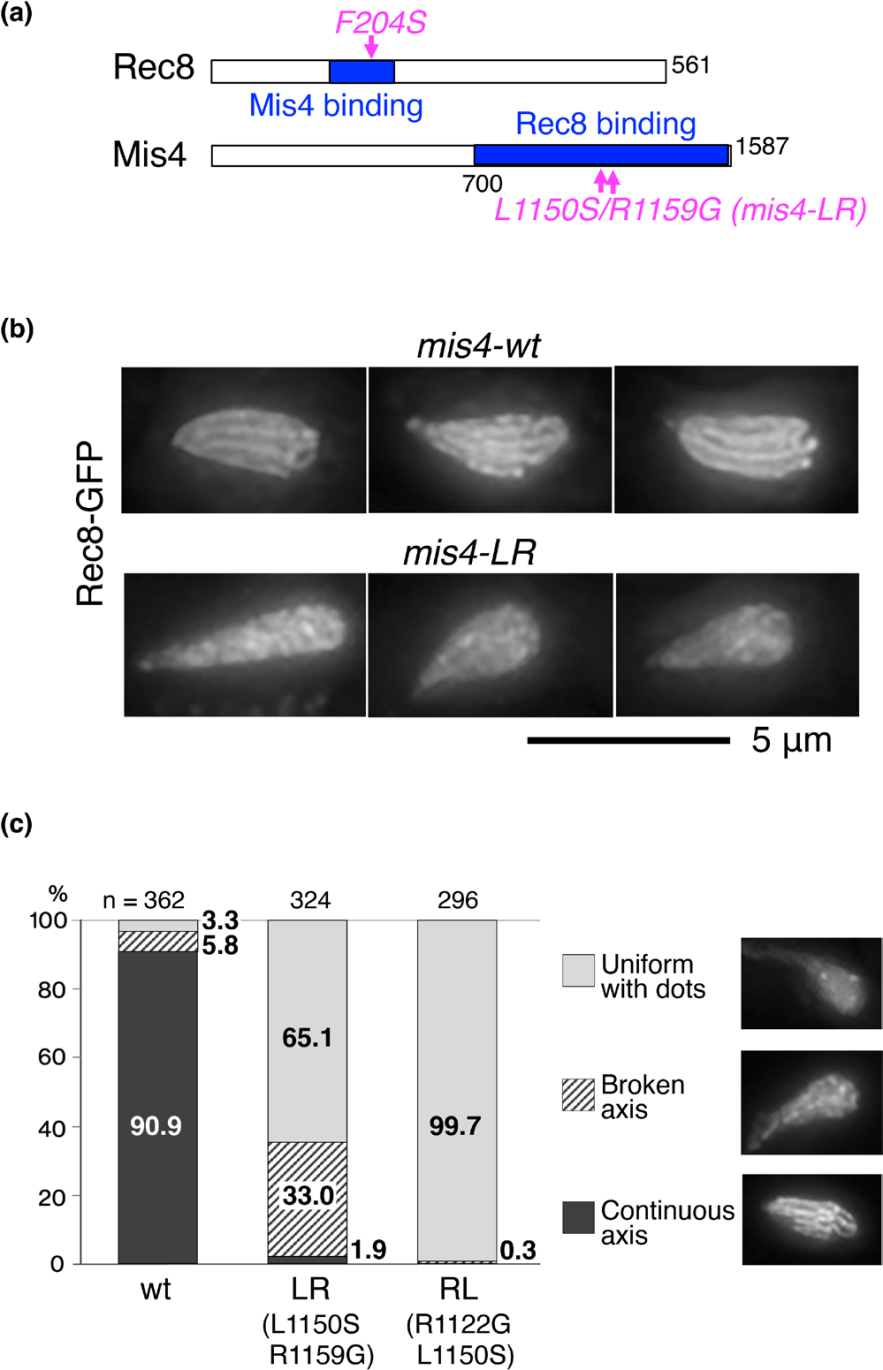
Axis morphology in *mis4* mutants. (a) Schematic diagram of Mis4 and Rec8. Mutation sites are indicated within the Mis4‐binding domain of Rec8 and Rec8‐binding domain of Mis4. (b) Fluorescence images of the chromatin axis visualized by Rec8‐GFP in wild‐type Mis4 (upper panels) and the Mis4‐LR mutant (lower panels) in a *mei4∆ wpl1∆* background. Larger fields of view containing additional cells are shown in Figure S2a‐c (wild type) and Figure S2d‐f (Mis4‐LR). (c) Axis morphology was categorized into three groups: Continuous axis, Broken axis, and Uniform with dots. Cells in each category were counted in at least three independent cultures. The numbers on the graph indicate the percentage of each category. The numbers of cells examined are shown at the top of the graph.

In a previous study, we demonstrated that a Rec8 mutant shows defects specifically in chromatin axis formation and homologous chromosome recombination without affecting sister chromatid cohesion. As the responsible mutation, F204S, resides within the Mis4‐binding domain of Rec8, the phenotype may arise from defective interaction between Rec8 and Mis4. Based on this idea, we attempted to identify *mis4* mutants that are specifically defective in axis formation without impairing cohesion function, similar to the *rec8‐F204S* mutant. To this end, we focused on the Rec8‐binding domain of Mis4 (Figure 1a). We used an *h* ^*90*^ mating‐type strain in a *mei4∆* background to arrest each transformant at meiotic prophase. We also introduced *wpl1∆* into the host strain to produce a prominent Rec8‐GFP axis (Figure S1a), allowing defects in chromatin axis formation to be readily evaluated by microscopic observation, as described previously (Sakuno et al. 2022). Using this strain as a screening host, randomly mutagenized *mis4* cDNA fragments encoding the C‐terminal region of Mis4 (amino acid residues 685–1587), generated by error‐prone PCR, were transformed. We screened for mutants that showed defects in axis formation while retaining normal cohesion function by fluorescence microscopy, based on Rec8‐GFP axis morphology.

### 2.2 Chromatin Axis Formation

Among approximately 3000 viable transformants in the *mis4* mutant library, we first identified two mutants, m8 and m24, that displayed defects in chromatin axis formation (Figure S1b) compared with *mis4* wild‐type cells (Figure 1b upper panels). The m8 mutant contained amino acid substitutions G1018R, L1150S, E1361G, and C1457R, whereas the m24 mutant contained R1122G and R1159G. We next examined the effect of each individual amino acid substitution. Among the four mutations in m8, only *mis4‐L1150S* showed defects in axis formation (Figure S1b). Among the two mutations in m24, both *mis4‐R1122G* and *mis4‐R1159G* showed little effect on the axis (Figure S1b). We then generated double mutants combining these substitutions. Among them, the double mutants *mis4‐L1150S/R1159G* (designated *mis4‐LR*) and *mis4‐R1122G/L1150S* (designated *mis4‐RL*) exhibited severe defects in axis formation (Figure 1b, Figure S2d‐f for *mis4‐LR*; Figure S1b for *mis4‐RL*). To further assess degrees of defects in chromatin axis morphology in these mutants, axis morphology was classified into three categories (continuous axis, broken axis, and uniform with dots), and their populations were counted in each mutant (Figure 1c). Wild‐type cells predominantly showed the continuous axis. In contrast, *mis4‐LR* cells showed increased populations of broken‐axis and uniform‐with‐dots categories, and *mis4‐RL* cells predominantly showed the uniform‐with‐dots category (Figure 1c). Thus, the LR mutation (L1150S/R1159G) confers severe defects in chromatin axis formation whereas the RL mutation (R1122G/L1150S) largely abolishes chromatin axis formation.

### 2.3 Homologous Recombination and Sister Chromatid Cohesion

**FIGURE 2:**
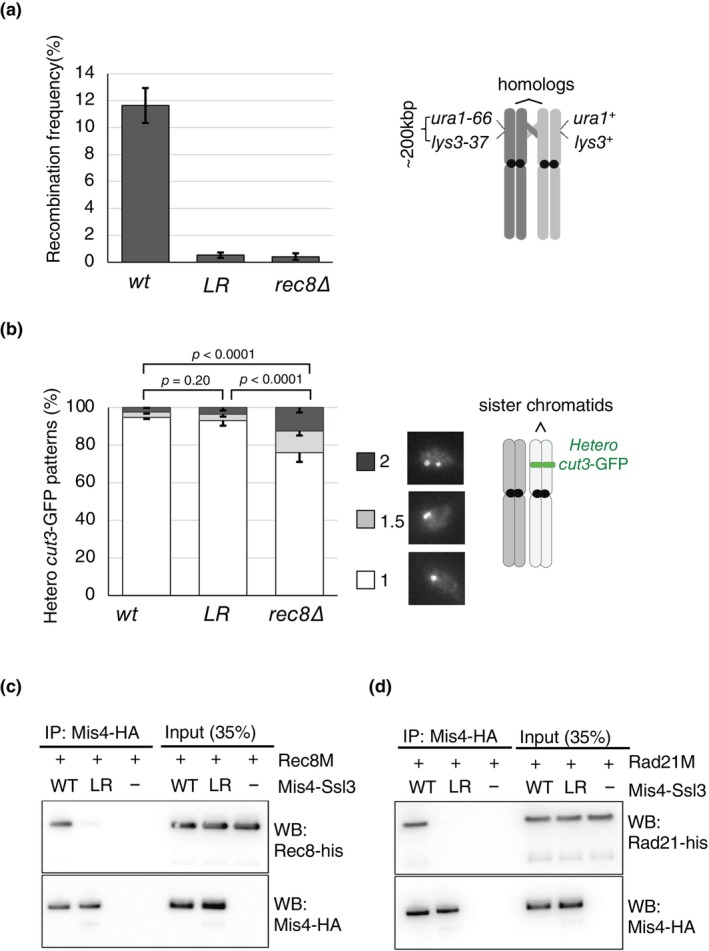
Characterization of *mis4* mutants. (a) Recombination frequency. Inset: Schematic of the assay. Recombination frequency between the *ura1* and *lys3* loci was measured. Recombination frequency (%) in wild‐type and Mis4‐LR cells. Data represent three independent experiments, with > 700 cells analyzed in each experiment. Error bars indicate standard deviation. (b) Sister chromatid cohesion. Inset: Schematic diagram of the assay, cohesion at the cut3‐GFP locus was evaluated by counting fluorescent foci (1, 1.5, or 2 per nucleus). Percentage of cells with each focus number in wild‐type and Mis4‐LR cells. Data represent three independent experiments, with > 130 cells analyzed in each experiment. Error bars indicate standard deviation. *p* values were obtained from chi‐square test for statistical significance. (c) Biochemical analysis of the Mis4 mutant protein. Co‐immunoprecipitation of wild‐type and mutant Mis4 with Rec8M (residues 104–450). Purified Mis4‐Ssl3 was incubated with His‐tagged Rec8M and immunoprecipitated using an anti‐HA antibody via a C‐terminal HA tag on Mis4. Rec8M and Mis4 in input and immunoprecipitated fractions were detected by western blotting with anti‐His or anti‐HA antibody, respectively. (d) Same as (c), except that His‐tagged Rad21M (residues 88–550) was used instead of Rec8M.

We next characterized the effects of these *mis4* mutations on recombination and sister chromatid cohesion. Homologous recombination and sister chromatid cohesion are both impaired in *rec8Δ* cells. Recombination frequency between the *ura1* and *lys3* loci was measured in these mutants. The recombination frequency was significantly reduced to a level comparable to that of *rec8Δ* in the double mutants *mis4‐LR* (Figure 2a) and *mis4‐RL* (Figure S3a), but was only moderately reduced in *mis4‐L1150S* (Figure S3a). We next examined sister chromatid cohesion using a *lacO* insertion at the *cut3* locus visualized by LacI‐GFP (cut3‐GFP), in a heterozygous strain. In this assay, cells with a single GFP focus represent intact sister chromatid cohesion, whereas those with two foci indicate loss of cohesion (Figure 2b). *mis4‐LR* cells showed a high proportion of single foci, similar to wild type, in contrast to *rec8Δ* cells (Figure 2b), indicating that sister chromatid cohesion is retained in *mis4‐LR*. In contrast, *mis4‐RL* cells showed an increased proportion of cells with two foci, indicating loss of sister chromatid cohesion (Figure S3b). Taken together, these results identify *mis4‐LR* (L1150S/R1159G) as a mutant specifically defective in chromatin axis formation while retaining normal cohesion function. Therefore, we focused on this mutant for further experiments.

### 2.4 Biochemical Analysis of the Purified Mis4‐LR Mutant Protein

*mis4‐LR* and *rec8‐F204S* mutants showed similar defects in chromatin axis formation during meiotic prophase, suggesting that the *mis4‐LR* mutations affect physical interaction with Rec8. To test this, we purified the Mis4‐Ssl3 complex and a truncated Rec8 lacking the N‐ and C‐termini (Rec8M) and confirmed their physical interaction using an in vitro pull‐down assay. Purified Mis4‐Ssl3 and Rec8M were incubated together, and Mis4‐Ssl3 was immunoprecipitated using a HA tag fused to Mis4. This resulted in co‐precipitation of Rec8M, detected via a histidine tag fused to its C‐terminus (Figure 2c). In contrast to wild‐type Mis4‐Ssl3, the Rec8M signal was markedly reduced when the LR mutant Mis4‐Ssl3 complex was used. We also found that interaction between Mis4 and Rad21 was weakened by the LR mutation (Figure 2d). These results indicate that Mis4 directly interacts with Rec8 and Rad21, and the LR mutations weaken interactions both with the meiotic cohesin Rec8 and mitotic cohesin Rad21.

### 2.5 Prediction of the Mis4‐Rec8 Interaction

**FIGURE 3:**
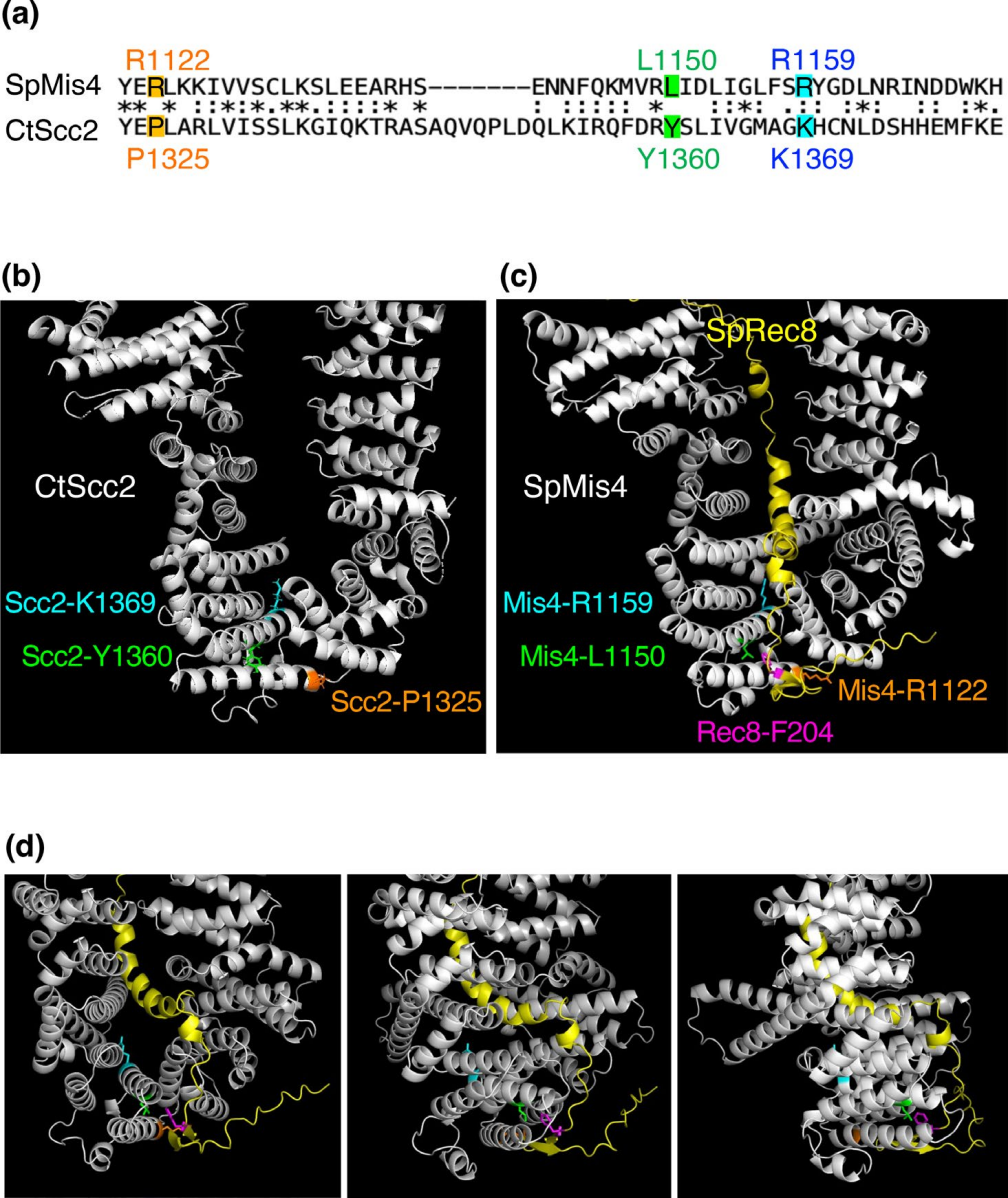
Interaction between Mis4 and Rec8. (a) Amino acid sequences of *S. pombe* Mis4 (SpMis4) and *C. thermophilus* Scc2 (CtScc2) spanning the mutation sites. (b) Structure of CtScc2 based on PDB entry 5T8V (Kikuchi et al. 2016). The C‐terminal region involved in Rec8 binding is shown. Residues of P1325, Y1360, and R1369 of CtScc2, corresponding to R1122, L1150, and R1159 of SpMis4, are indicated by orange, green, and blue, respectively. (c) Structure of SpMis4 and SpRec8 predicted by AlphaFold 3. The Rec8‐binding domain of SpMis4 (residues 700–1587) and the Mis4‐binding domain of SpRec8 (residues 141–280) are shown in white and yellow, respectively. Residues Mis4‐R1122, Mis4‐L1150, Mis4‐R1159, and Rec8‐F204 are shown in orange, green, blue, and magenta, respectively. (d) Views of (c) from different rotation angles: 30°, 60°, and 90° around the y‐axis, from left to right. The same color scheme as in (c) is used. A rotating model is shown in Supporting Information Movie S1.

The crystal structure of Scc2 (a homolog of Mis4) has been determined in the thermophilic fungus *Chaetomium thermophilum* (Kikuchi et al. 2016); this protein (CtScc2) adopts a U‐shaped structure composed of α‐helix domains. Amino acid sequences spanning the mutation sites are shown for CtScc2 and *S. pombe* Mis4 (SpMis4) (Figure 3a). The crystal structure of CtScc2 was generated from the atomic coordinates of PDB 5T8V (Kikuchi et al. 2016), and Figure 3b shows its C‐terminal region, which contains the predicted Rec8‐binding region.

We predicted the structure of SpMis4 using AlphaFold3 and obtained a U‐shaped structure similar to that of CtScc2, in which the Mis4‐binding domain of Rec8 is positioned between the U‐shaped arms of Mis4 (Figure 3c). The mutation sites Mis4‐R1122, Mis4‐L1150, Mis4‐R1159, and Rec8‐F204 are all located within the cleft formed by the U‐shaped domains (Figure 3c,d). Correspondingly, P1325, Y1360, and K1369 in CtScc2 (equivalent to R1122, L1150, and R1159 in SpMis4) are also located in this cleft (Figure 3b). This predicted model of Mis4 and Rec8 also supports the potential significance of the Mis4‐Rec8 interaction.

## 3 Discussion

Meiotic cohesin Rec8 functions in both sister chromatid cohesion and homologous chromosome recombination. Our studies have demonstrated that *rec8‐F204S* and *mis4‐LR* mutants separate these closely associated functions (Sakuno et al. 2022; this study). A recent study reported a human cohesin mutant with the opposite phenotype, that is, defective in sister chromatid cohesion but normal in DNA loop formation (Nagasaka et al. 2023). These observations suggest that sister chromatid cohesion and DNA loop formation are mediated by distinct mechanisms.

### 3.1 Possible Mechanisms for Functional Specificity of the Mutations

Mis4 promotes cohesin loading onto DNA via its DNA‐binding activity and interactions with cohesin, concomitant with activation of cohesin's ATPase (Higashi et al. 2020; Kurokawa and Murayama 2020). Mis4 binds to DNA via two distinct α‐helix domains, both of which are located distantly from the mutations found in this study (Kurokawa and Murayama 2020). Therefore, these mutations of Mis4 (R1122G, L1150S, and R1159G) are not expected to affect its DNA binding.

The *mis4‐LR* mutations (L1150S/R1159G) weaken interaction with Rec8 but retain sister chromatid cohesion. DNA loop formation, which underlies chromatin axis‐loop formation, likely requires a stable interaction between Rec8 and Mis4. Weakened Mis4‐Rec8 interaction may permit DNA entrapment by cohesin sufficient to mediate a basal level of sister chromatid cohesion in both mitosis and meiosis, but is insufficient to support DNA loop formation in meiosis. When combined with L1150S, the R1122G mutation causes defects in sister chromatid cohesion, and axis formation defects more severe than R1159G (compare *mis4‐RL* with *mis4‐LR*; Figure 1c, Figure S3b). These results suggest that the R1122 residue of Mis4 plays a more significant role in cohesin function than R1159. Broken axis observed in *mis4‐LR* (Figure 1c) may be sufficient for sister chromatid cohesion, but insufficient for efficient homologous recombination. This is also consistent with the fact that *mis4‐LR* causes defects in meiosis involving Rec8, but not in mitosis, which involves Rad21. However, we cannot exclude the possibility that meiotic phenotypes may be influenced by chromatin structure produced with Rad21 in vegetative cells. The functional specificity of these mutations could be elucidated through further investigation of mutants affecting DNA binding, ATPase activities, and cohesin binding.

### 3.2 Biological Significance of the Mis4‐Rec8 Interaction

We have identified Mis4 and Rec8 mutations that affect cohesin functions and mapped these mutation sites to the interaction surface between Mis4 and Rec8. This Mis4‐Rec8 interaction surface is also conserved in the interaction between human NIPBL and Rad21 (Shi et al. 2020). Furthermore, NIPBL mutations associated with Cornelia de Lange syndrome occur within this interaction surface and impair cohesin‐mediated DNA loop extrusion (Panarotto et al. 2022). Therefore, these residues of NIPBL/Mis4 play an important role in modulating cohesin function through interactions with Rec8 and Rad21.

## 4 Experimental Procedures

### 4.1 *Schizosaccharomyces pombe* Strains

All media and growth conditions were as described previously (Moreno et al. 1991). Complete medium supplemented with uracil, lysine, adenine, histidine, and leucine (YE5S), minimal medium (EMM2), and sporulation‐inducing medium (SPA and SSA) were used unless otherwise stated. The strains used in this study are listed in Table S1.

Deletion of endogenous genes and tagging with 3 × flag tag were performed using a PCR‐based gene targeting method for *S. pombe*, using the *hphMX6* (*hygR*), *natMX6* (*natR*), and *bsdR* genes as selection markers. To generate strains of *mis4* point mutations with a 3 × flag tag, the coding region corresponding to the C‐terminal 685 amino acids of *mis4+* (OLI3462), together with a sequence extending 528 bp downstream of the stop codon (OLI3202), was amplified by colony PCR using *mis4*‐3 × flag‐tagged strain (YS119) as a template. The amplified fragment was cloned into pUC119 using the NEBuilder system (New England Biolabs) to generate pUC119‐mis4C‐wt‐3flag‐nat‐3UTR. Subsequently, additional plasmids were constructed in which the selection marker of pUC119‐mis4C‐wt‐3flag‐nat‐3UTR was replaced with either hyg or bsd (pUC119‐mis4C‐wt‐3flag‐hyg‐3UTR or pUC119‐mis4C‐wt‐3flag‐bsd‐3UTR). Target sequences of *mis4* ^*+*^ were then mutated using the KOD One Master Mix Blue (TOYOBO) to introduce amino acid substitutions. The C‐terminal portion of the *mis4* ^*+*^ open reading frame, including the mutation site and marker gene with the *mis4* ^*+*^ terminator, was amplified by PCR and transformed into the wild‐type *mis4* ^*+*^ locus. Chromosomal integration of the mutated fragment was selected by the appropriate marker, and correct replacement was confirmed by PCR and direct sequencing.

### 4.2 Mis4 Mutant Screening

Mutations were introduced into the Rec8‐binding domain (amino acid residues 685–1587) of Mis4 by error‐prone PCR (Figure S1a). Error‐prone PCR of *mis4* ^*+*^ cDNA in pUC119‐mis4C‐wt‐3flag‐nat‐3UTR by Ex Taq DNA Polymerase (TaKaRa Bio) was performed using multiple conditions: varying MnCl_2_ concentrations (final 0.05, 0.15, 0.25, and 0.3 mM) and the addition of 1 mM dGTP or dATP to 0.2 mM of dNTP mix (eight conditions in total), using a reverse primer containing the 3 × flag sequence (OLI3198). In parallel, a PCR fragment containing the 3 × flag sequence, the nat resistance marker *natMX6* (*natR*), and the *mis4+* terminator was amplified from pUC119‐mis4C‐wt‐3flag‐nat‐3UTR. These fragments were assembled by PCR using KOD One Master Mix Blue (TOYOBO), with the flag sequence serving as an overlapping region. The resulting PCR products (*mis4* mutant library) were used to transform host strains for screening. The *h* ^*90*^ mating‐type strain in the *mei4∆ wpl1∆* background was used as the host strain: *mei4∆* to arrest each transformant at meiotic prophase on sporulation medium plates (Horie et al. 1998), and *wpl1∆* to produce a prominent Rec8‐GFP axis (Sakuno et al. 2022). More than 95% of blasticidin‐resistant colonies obtained after transformation showed correct integration, as confirmed by PCR. Rec8‐GFP signals during meiotic prophase were examined in colonies that exhibited relatively normal growth, thereby generating a *mis4* mutant library suitable for screening.

### 4.3 Preparation of Meiotic Cells

For microscopic observation of Rec8‐GFP or Rec8‐GFP, *h* ^*90*^ cells cultured on YE plates at 26°C overnight were streaked on SSA plates or suspended in 20 mg/mL of leucine and spotted on SPA or SSA plates. Cells were incubated at 26°C for 6–10 h to observe the horsetail stage.

For *lacO*/lacI‐GFP‐based cohesion assays, *h* ^*+*^ and *h* ^*−*^ cells were mixed in the presence of 20 mg/mL leucine and spotted onto SSA or SPA plates. Diploid cells were grown in EMM2 liquid medium containing 5 mg/mL NH_4_Cl at a density of 5 × 10^6^ cells/mL at 25°C, then resuspended in EMM2 (1% glucose) medium lacking NH_4_Cl at a density of 1 × 10^7^ cells/mL and cultured at 25°C for 15 h.

### 4.4 Fluorescence Microscopy

Fluorescence images were obtained using an Axioplan2 microscope (Carl Zeiss) equipped with a Quantix cooled CCD camera (Photometrics) and a DeltaVision microscope system (GE Healthcare Inc.) equipped with a CoolSNAP HQ^2^ cooled CCD camera (Photometrics) and a 60× Plan‐ApoN SC oil immersion objective lens (NA = 1.40; Olympus). The brightness of the images was adjusted using Fiji software without changing the gamma settings.

### 4.5 Recombination Assay

For intergenic recombination analysis, haploid strains carrying appropriate markers were mated and sporulated at 26°C on SSA plates for 30 h. Spores were isolated using β‐glucuronidase solution (Wako) and plated on YE medium. Colonies were then replica‐plated onto selective media [EMM2‐uracil, EMM2‐lysine, EMM2‐lysine, YE5S containing hygromycin B (FUJIFILM) or YE5S containing clonNAT (WERNER BioAgents)] and YE5S containing phloxine B (Wako) to select haploid cells. Recombination frequency in the *ura1‐lys3* interval was determined by assessing lysine auxotrophy among uracil prototrophic colonies (*n* > 700, replicated three times).

### 4.6 Immunoprecipitation

Wild‐type Mis4 and the Mis4‐LR mutant (L1150S/R1159G) proteins were expressed in *S. pombe* cells and purified as heterodimer complexes with Ssl3 (Mis4‐Ssl3), as described previously (Murayama and Uhlmann 2014). *S. pombe* Rad21M (residues 88–550, fused to a C‐terminal 6 × His tag) was expressed in *E. coli* and purified as described previously (Kurokawa and Murayama 2020). To purify Rec8M (residues 104–450), the corresponding cDNA was fused to a GST tag at the N‐terminus and a 7 × His tag at the C‐terminus and cloned into the pGEX6P plasmid. The plasmid was introduced into *E. coli* strain Rosetta 2 (DE3) and cultured in 1 L LB‐Miller broth to OD_600_ = 0.5 at 30°C. Isopropyl β‐D‐1‐thiogactopyranoside (IPTG) was added to a final concentration of 0.5 mM, and cells were further cultured for 2 h at 30°C. Cells were resuspended in R buffer (25 mM Tris–HCl, pH 7.5, 10% (v/v) glycerol) containing 0.3 M NaCl, 0.01% (w/v) NP‐40, and EDTA‐free cOmplete protease inhibitor cocktail (Merck) and disrupted by sonication. After clarification by centrifugation at 280,000 × *g* for 30 min, the lysate (50 mL) was incubated with 1 mL bed volume of Glutathione Sepharose 4 Fast Flow resin (Cytiva) for 1 h. The resin was washed with 80 mL of R buffer containing 0.3 M NaCl and 0.01% (w/v) NP‐40 and bound proteins were eluted with 25 mM glutathione in the same buffer, followed by purification using 0.5 mL bed volume of NiNTA metal affinity resin (Qiagen). After 1‐h incubation, the resin was washed with 10 mL of R buffer containing 0.3 M NaCl and 0.01% (w/v) NP‐40. The resin was resuspended in 5 mL of the same buffer containing 5 mM ATP and 5 mM MgCl_2_ and incubated for 30 min. The resin was further washed with 10 mL of R buffer containing 0.3 M NaCl. Bound proteins were eluted with 200 mM imidazole in the same buffer. After concentration by ultrafiltration (Amicon Ultra‐4, Y‐10, Merck), the eluate was dialyzed against R buffer containing 250 mM NaCl and 0.5 mM tris(2‐carboxyethyl) phosphine (TCEP), and the aliquots were flash‐frozen by liquid nitrogen and stored at −80°C.

Purified Mis4‐Ssl3 (150 nM) was incubated with Rec8M (150 nM) or Rad21M (180 nM) in IP buffer (35 mM Tris–HCl, pH 7.5, 100 mM NaCl, 1 mM MgCl_2_, 0.01% (w/v) Tween 20 and 5% (v/v) glycerol) at 30°C for 10 min. Anti‐HA bound protein A‐conjugated magnetic beads (Thermo Fisher) were added and incubated at 4°C for 1 h to immunoprecipitate Mis4‐Ssl3 via the HA tag. Beads were washed twice with IP buffer, and bound proteins were analyzed by SDS‐PAGE, followed by western blotting.

### 4.7 Prediction of Structure and Interaction of Proteins

The structure and interaction between Mis4 and Rec8 were predicted using AlphaFold 3 (Abramson et al. 2024). Coordinates were visualized using PyMOL (version 3.0.3). The Rec8‐binding domain of Mis4 (amino acid residues 700–1587) and the Mis4‐binding domain of Rec8 (amino acid residues 141–280) are shown in Figure 1c.

## Author Contributions

**Yasuto Murayama:** methodology, investigation, validation, formal analysis, writing – review and editing, writing – original draft, visualization, funding acquisition. **Takeshi Sakuno:** conceptualization, methodology, data curation, investigation, validation, formal analysis, resources, writing – review and editing, writing – original draft. **Yasushi Hiraoka:** conceptualization, methodology, data curation, validation, formal analysis, supervision, investigation, funding acquisition, writing – review and editing, writing – original draft, resources, visualization. **Tokuko Haraguchi:** conceptualization, formal analysis, supervision, funding acquisition, resources, writing – original draft, writing – review and editing, validation.

## Funding

This work was supported by Japan Society for the Promotion of Science (JP23K05636, JP18H05528, JP24H02282, JP25H00989).

## Conflicts of Interest

The authors declare no conflicts of interest.

## Supporting information


**Figure S1:** Screening of mis4 mutants.
**Figure S2:** Fluorescence image of Rec8‐GFP in wildtype and LR mutant cells.
**Figure S3:** Characterization of mis4 mutants.
**Table S1:** List of *S. pombe* strains used in this study.

**Movie S1:** A rotating model of the Mis4‐Rec8 interaction surface.

## Data Availability

The data that support the findings of this study are available from the corresponding author upon reasonable request.
