# Supplementary material for: Interaction Between Rec8 and Mis4 Is Required for Axis‐Loop Chromatin Formation and Homologous Chromosome Recombination During Meiosis
Source: Genes Cells. 2026 Jun 10;31(4):e70128. doi: 10.1111/gtc.70128 (PMC13253173; doi:10.1111/gtc.70128)
Supplement: Supplementary file 1 — Figure S1: Screening of mis4 mutants. Figure S2: Fluorescence image of Rec8‐GFP in wildtype and LR mutant cells. Figure S3: Characterization of mis4 mutants. Table S1: List of S. pombe strains used in this study. [file GTC-31-0-s001.pdf]

Supplementary information

Interaction between Rec8 and Mis4 is required for axis-loop chromatin formation and homologous chromosome recombination during meiosis

Takeshi Sakuno[1], Yasuto Murayama[2], Tokuko Haraguchi[1], and Yasushi Hiraoka[1]*

[1] Graduate School of Frontier Biosciences, The University of Osaka, Suita 565-0871, Japan; [2] National Institute of Genetics, Mishima 411-8540, Japan

Figure S1. Screening of *mis4* mutants.

Figure S2. Fluorescence image of Rec8-GFP in wildtype and LR mutant cells.

Figure S3. Characterization of *mis4* mutants.

Table S1. List of *S. pombe* strains used in this study.

Movie S1. A rotating model of the Mis4-Rec8 interaction surface.

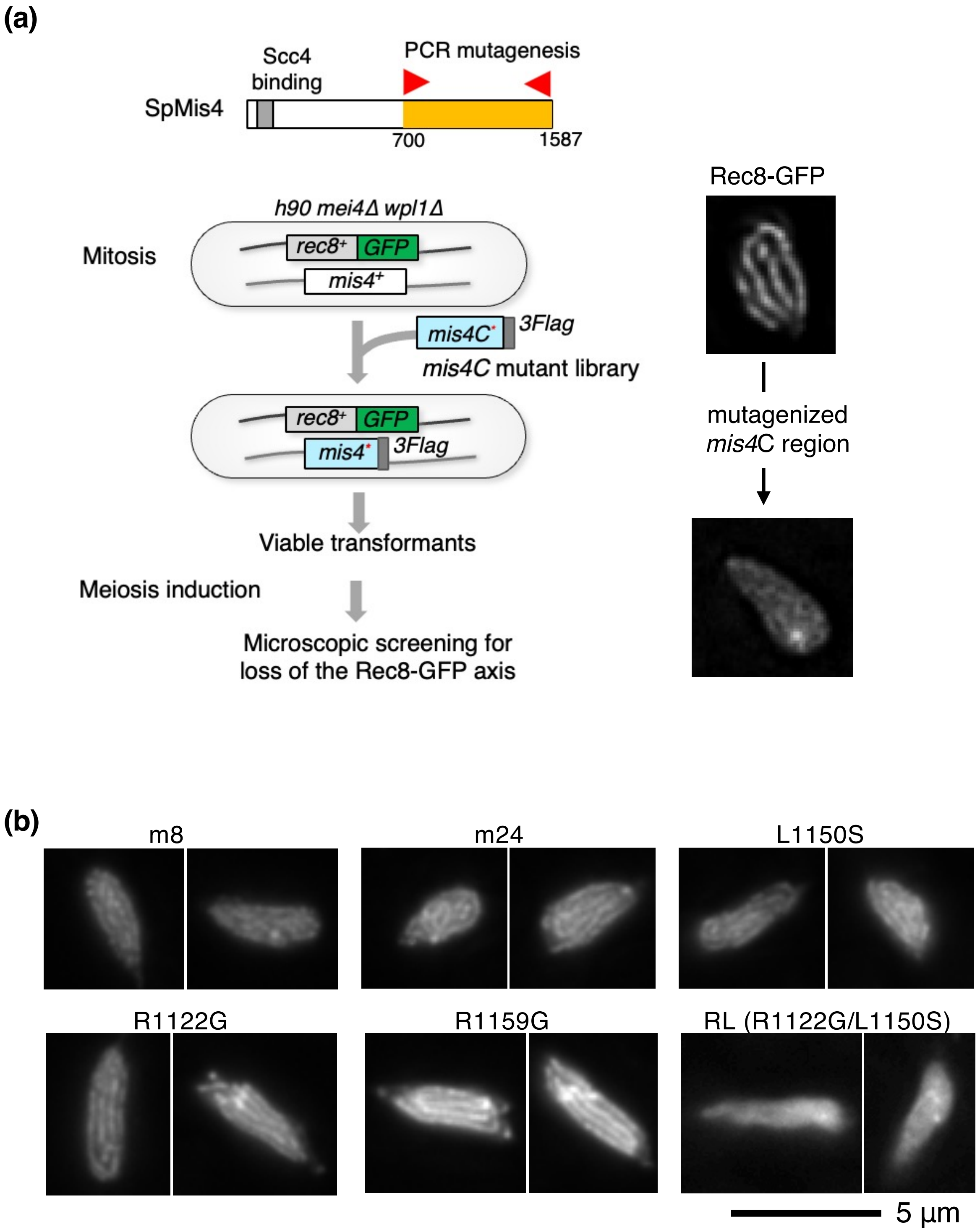

Figure S1. Screening of *mis4* mutants.

(a) Strategy of mutant screening (left). Mutations were introduced into the Rec8-binding region of Mis4 by error-prone PCR. The resulting fragments (*mis4* mutant library) were used to transform host cells (*h^{90} mei4*Δ *wpl1*Δ expressing Rec8-GFP). Chromosomal integration was selected using a selection marker, and mutants were screened by fluorescence microscopy of Rec8-GFP. Representative images (right): the host strain displays a normal chromatin axis (upper), whereas mutants defective in chromatin axis formation were selected (lower). **(b)** Fluorescence microscope images of Rec8-GFP in *mis4* mutants. Two representative cells are shown for each mutant: m8, m24, L1150S, R1122G, R1159G, and R1122G/L1150S.

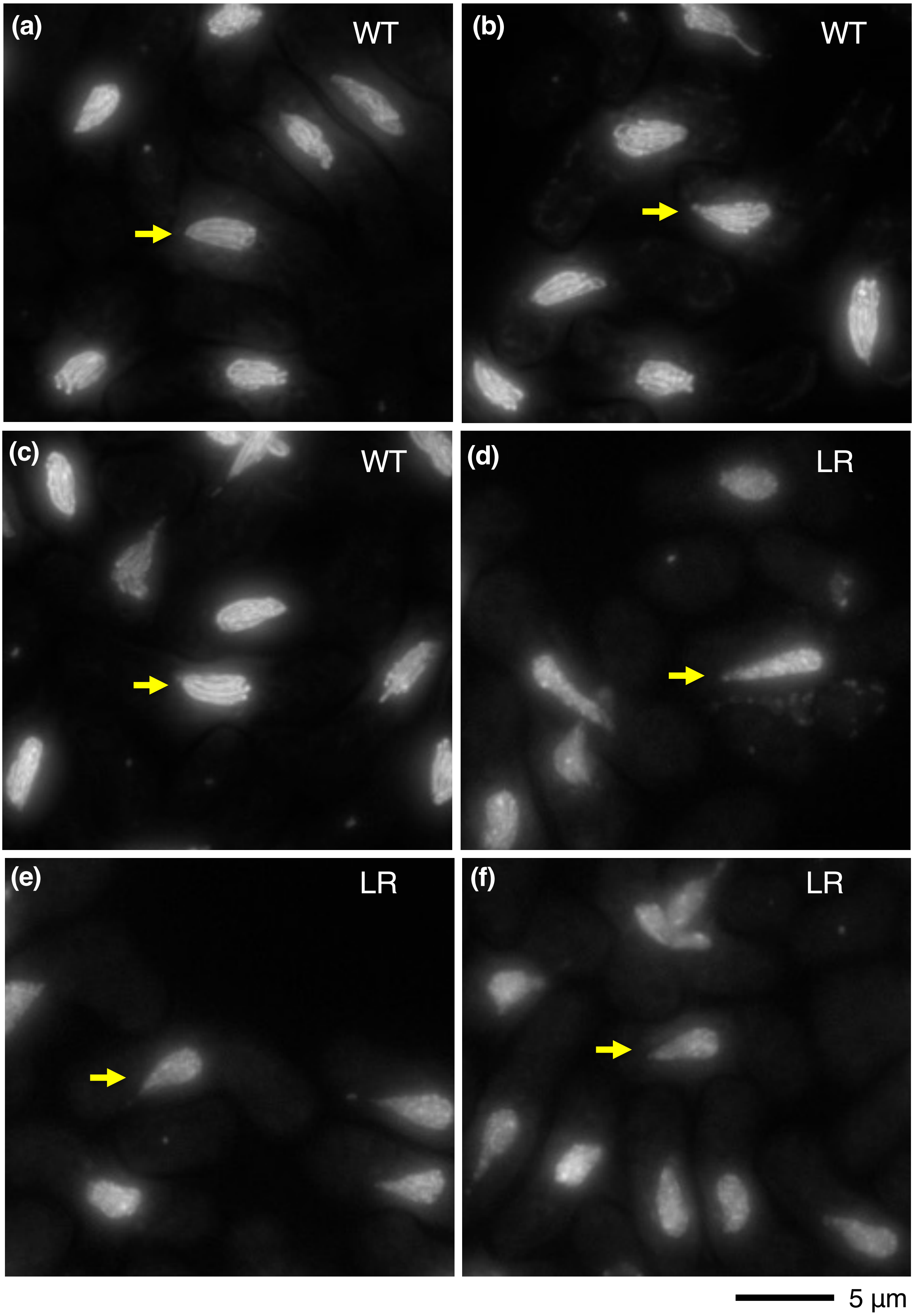

Figure S2. Fluorescence image of Rec8-GFP in wildtype and LR mutant cells. Larger fields of view of Figure 1b for *mis4* wildtype **(a-c)** and *mis4-LR* mutant **(d-f)**. Arrows indicate the cells shown in the corresponding panels of Figure 1b: (**a-c**) upper left, middle, and right, respectively; (**d-f**) lower left, middle, and right, respectively.

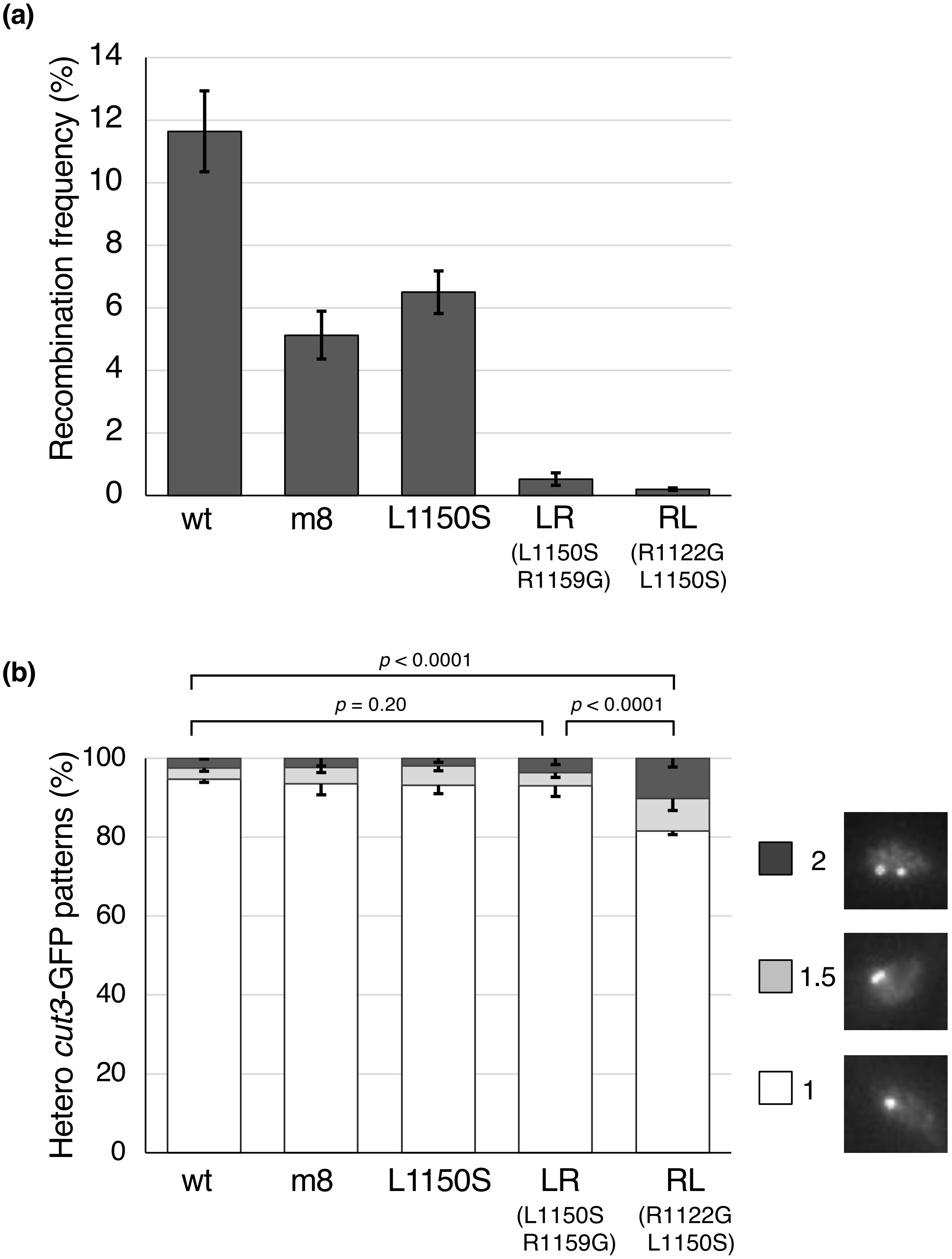

Figure S3. Characterization of *mis4* mutants.
(a) Recombination frequency between *ura1* and *lys3* loci in the indicated strains. Data represent three independent experiments, with >700 cells analyzed in each experiment. Error bars indicate standard deviation. **(b)** Sister chromatid cohesion. The heterozygous *cut3* locus was visualized using the *lacO*/LacI-GFP system. One focus indicates cohesion, whereas two foci indicate separation. Data represent three independent experiments, with >130 cells analyzed in each experiment. Error bars represent standard deviation. *p* values were obtained from chi-square test for statistical significance.

Table S1. List of *S. pombe* strains used in this study

Strain name	Mating type	Genotype	Source	Figure numbers
YS1140	*h90*	*mis4-3flag-nat rec8-GFP-kanr Δmei4::ura4 Δwpl1::HB*	This study	1b, 1c, S2
YS1170	*h90*	*mis4-L1150S R1159G-3flag-nat rec8-GFP-kanr Δmei4::ura4 Δwpl1::HB*	This study	1b, 1c, S2
YS1144	*h90*	*mis4-m8-3flag-nat rec8-GFP-kanr Δmei4::ura4 Δwpl1::HB*	This study	S1b
YS1168	*h90*	*mis4-L1150S-3flag-nat rec8-GFP-kanr Δmei4::ura4 Δwpl1::HB*	This study	S1b
YS1148	*h90*	*mis4-m24-3flag-nat rec8-GFP-kanr Δmei4::ura4 Δwpl1::HB*	This study	S1b
YS1171	*h90*	*mis4-R1122G-3flag-nat rec8-GFP-kanr Δmei4::ura4 Δwpl1::HB*	This study	S1b
YS1174	*h90*	*mis4-R1159G-3flag-nat rec8-GFP-kanr Δmei4::ura4 Δwpl1::HB*	This study	S1b
YS1169	*h90*	*mis4-L1150S R1122G-3flag-nat rec8-GFP-kanr Δmei4::ura4 Δwpl1::HB*	This study	1c, S1b
YS1217	*h+*	*mis4-wt-3flag-nat ura1 lys3*	This study	2a, S3a
YS1366	*h-*	*mis4-3flag-nat leu1*	This study	2a, S3a
YS1226	*h+*	*mis4-L1150S R1159G-3flag-nat ura1 lys3*	This study	2a, S3a
YS1374	*h-*	*mis4-L1150S R1159G-3flag-nat leu1*	This study	2a, S3a
YS561	*h+*	*Δrec8::kanr leu1 ade6-M216*	This study	2a, S3a
YS1276	*h-*	*Δrec8::kanr ura1 lys3*	This study	2a, S3a
YS1229	*h+*	*mis4-m8-3flag-nat ura1 lys3*	This study	S3a
YS1129	*h-*	*mis4-m8-3flag-nat leu1*	This study	S3a
YS1220	*h+*	*mis4-L1150S-3flag-nat ura1 lys3*	This study	S3a
YS1232	*h-*	*mis4-L1150S-3flag-nat leu1*	This study	S3a
YS1223	*h+*	*mis4-L1150S R1122G-3flag-nat ura1 lys3*	This study	S3a
YS1370	*h-*	*mis4-L1150S R1122G-3flag-nat leu1*	This study	S3a
YS1245	*h-*	*cut3-lacO his7+::Pdis1-GFP-lacI rec8-wt-3Pk-bsd mis4-wt-3flag-nat leu1 Δmei4::ura4*	This study	2b, S3b
YS1219	*h+*	*mis4-wt-3flag-nat Δmei4::ura4*	This study	2b, S3b
YS1251	*h-*	*cut3-lacO his7+::Pdis1-GFP-lacI rec8-wt-3Pk-bsd mis4-L1150S R1159G-3flag-nat leu1 Δmei4::ura4*	This study	2b, S3b
YS1228	*h+*	*mis4-L1150S R1159G-3flag-nat Δmei4::ura4*	This study	2b, S3b
YS646	*h-*	*cut3-lacO his7+::Pdis1-GFP-lacI Δrec8::kanr leu1 Δmei4::ura4*	This study	2b, S3b
PZ416	*h+*	*Δrec8::kanr Δmei4::kanr ade6*	Yamamoto Lab	2b, S3b
YS1253	*h-*	*cut3-lacO his7+::Pdis1-GFP-lacI rec8-wt-3Pk-bsd mis4-m8-3flag-nat leu1 Δmei4::ura4*	This study	S3b
YS1231	*h+*	*mis4-m8-3flag-nat Δmei4::ura4*	This study	S3b
YS1247	*h-*	*cut3-lacO his7+::Pdis1-GFP-lacI rec8-wt-3Pk-bsd mis4-L1150S-3flag-nat leu1 Δmei4::ura4*	This study	S3b
YS1222	*h+*	*mis4-L1150S-3flag-nat Δmei4::ura4*	This study	S3b
YS1249	*h-*	*cut3-lacO his7+::Pdis1-GFP-lacI rec8-wt-3Pk-bsd mis4-L1150S R1122G-3flag-nat leu1 Δmei4::ura4*	This study	S3b
YS1266	*h+*	*mis4-L1150S R1122G-3flag-nat Δmei4::ura4 leu1*	This study	S3b
YST37	*h-*	*pREP1-mis4-wt-HA-PreScission site-2x protein A, pREP2-ssl3-wt, leu1-32, ura4-D18*	This study	2c, 2d
YST482	*h-*	*pREP1-mis4-L1150S R1122G-HA-PreScission site-2x protein A, pREP2-ssl3-wt, leu1-32, ura4-D18*	This study	2c, 2d
